# Supplementary material for: Declines in skeletal muscle quality vs. size following two weeks of knee joint immobilization
Source: PeerJ. 2020 Jan 13;8:e8224. doi: 10.7717/peerj.8224 (PMC6964688; doi:10.7717/peerj.8224)

**Post-hoc power analysis – VL Echo Intensity following two weeks of immobilization – left limb**


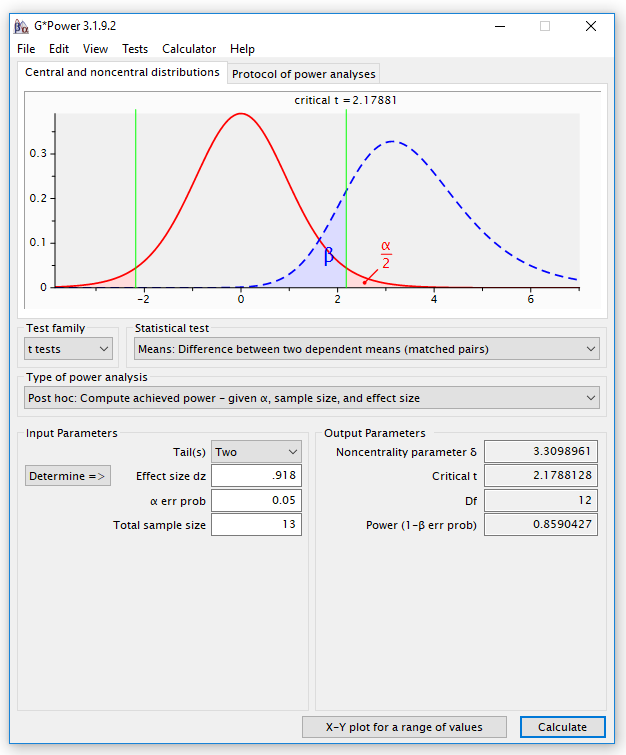


**Post-hoc power analysis – VL CSA following two weeks of immobilization – left limb**


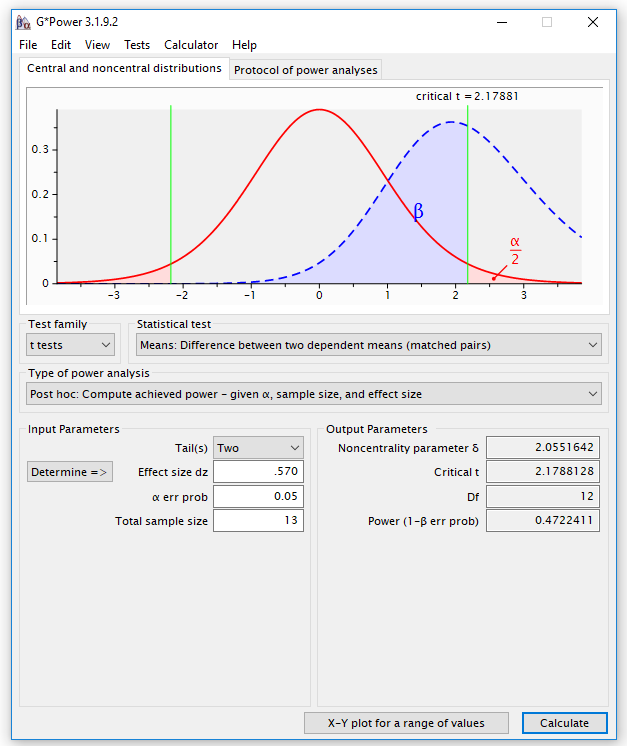


**Post-hoc power analysis – RF Echo Intensity following two weeks of immobilization – left limb**


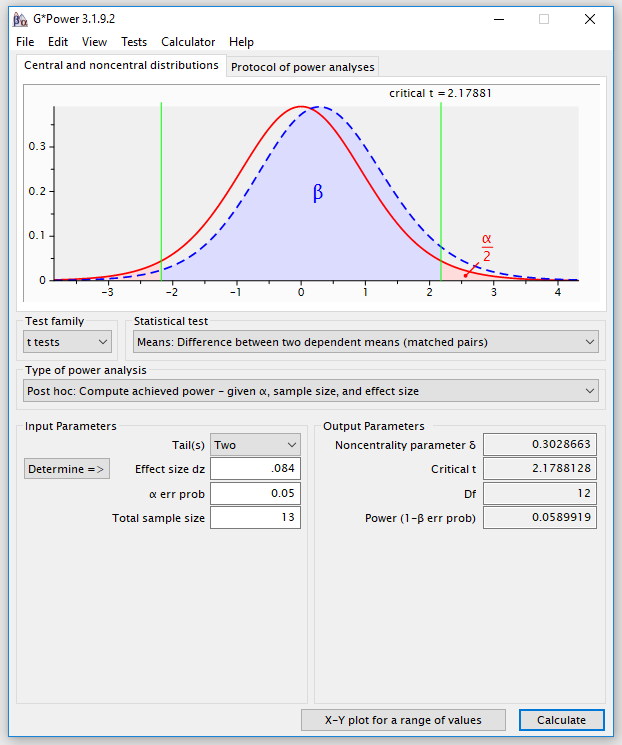


**Post-hoc power analysis – RF CSA following two weeks of immobilization – left limb**


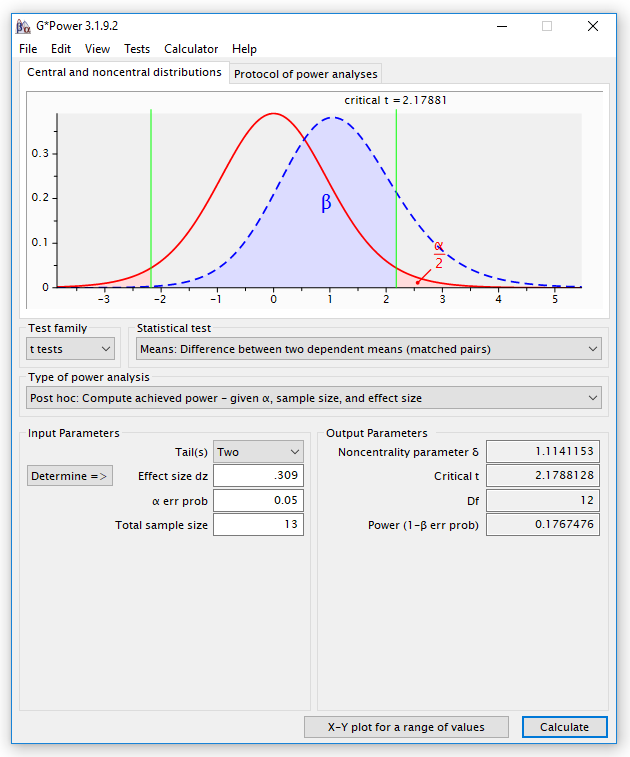

Supplement: Supplemental Information 2 — G*Power (version 3.1.9.2, Heinrich-Heine-Universität Düsseldorf) post-hoc power analyses for echo intensity and cross-sectional area of the immobilized knee extensors. For additional information, the reader is directed to the work of Beck (2013). [file peerj-08-8224-s002.docx]
